# Fast photosynthesis measurements for phenotyping photosynthetic capacity of rice

**DOI:** 10.1186/s13007-020-0553-2

**Published:** 2020-01-24

**Authors:** Tingting Du, Ping Meng, Jianliang Huang, Shaobing Peng, Dongliang Xiong

**Affiliations:** National Key Laboratory of Crop Genetic Improvement, MOA Key Laboratory of Crop Ecophysiology and Farming System in the Middle Reaches of the Yangtze River, Huazhong Agricultural University, Wuhan, 430070 Hubei China

**Keywords:** Phenotyping, Photosynthetic capacity, Gas exchange, Genetic variation, Light response curve, Rice

## Abstract

**Background:**

Over the past decades, the structural and functional genomics of rice have been deeply studied, and high density of molecular genetic markers have been developed. However, the genetic variation in leaf photosynthesis, the most important trait for rice yield improvement, was rarely studied. The lack of photosynthesis phenotyping tools is one of the bottlenecks, as traditional direct photosynthesis measurements are very low-throughput, and recently developed high-throughput methods are indirect measurements. Hence, there is an urgent need for a fast, accurate and direct measurement approach.

**Result:**

We reported a fast photosynthesis measurement (FPM) method for phenotyping photosynthetic capacity of rice, which measures photosynthesis of excised tillers in environment-controlled lab conditions. The light response curves measured using FPM approach coped well with that the curves measured using traditional gas exchange approach. Importantly, the FPM technique achieved an average throughput of 5.4 light response curves per hour, which was 3 times faster than the 1.8 light response curves per hour using the traditional method. Tillers sampled at early morning had the highest photosynthesis, stomatal conductance and the lowest variability. In addition, even 12 h after sampling, there was no significant difference of photosynthesis rate between excised tillers and in situ. We finally investigated the genetic variations of photosynthetic traits across 568 F2 lines using the FPM technique and discussed the logistics of screening several hundred samples per day per instrumental unit using FPM to generate a wealth of photosynthetic phenotypic data, which might help to improve the selection power in large populations of rice with the ultimate aim of improving yield through improved photosynthesis.

**Conclusions:**

Here we developed a high-throughput method that can measure the rice leaf photosynthetic capacity approximately 10 times faster than traditional gas exchange approaches. Importantly, this method can overcome measurement errors caused by environmental heterogeneity under field conditions, and it is possible to measure 12 or more hours per day under lab conditions.

## Background

Crop yields must increase dramatically to feed the growing global population [19, 22]. Carbon assimilation rate is a major productivity-related trait that has yet to be improved [6, 24, 47], and the overarching reason is our inability to efficiently screen large numbers of plants [29, 34]. To meet the rapid growth of global food requirement, high-throughput phenotyping of photosynthesis method must be developed. Recently, some high-throughput approaches, such as thermal imagery, hyperspectral reflectance, chlorophyll fluorescence, normalized difference vegetation index (NDVI) and infrared thermography, have been developed for studying photosynthesis [8, 12, 33]. However, these approaches are typically indirect photosynthesis measurements, and some of them are challenging to operate in the field. As the relationships between photosynthetic indexes based on indirect approaches and photosynthetic rate are far from “direct”, these approaches require calibration and validation against direct photosynthesis measurements [28].

**Fig. 1:**
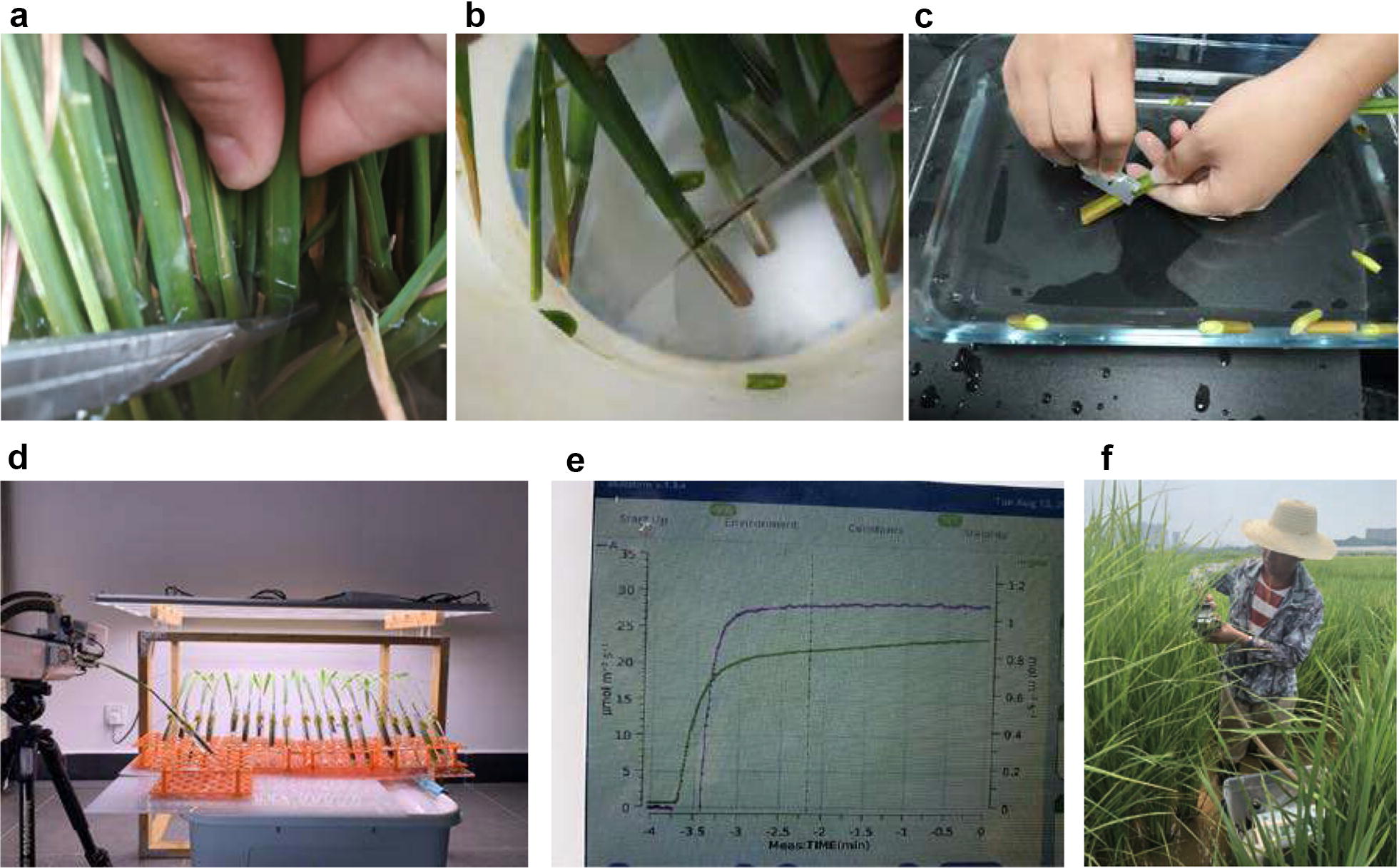
Photographs of the FPM technique (a-e) and gas exchange measurements in a flooded field (f). **a** Tillers were first cut under field water; **b** tillers were immediately transferred to airless distilled water, and a second-cut was created under water; **c** tillers were re-cut under airless distilled water after removing all the non-target region; **d** leaves were illuminated using a LED light source before performing gas exchange measurement (photosynthetic photo flux density = 1500 μmol m^−2^ s^−1^); **e** sample the fast gas exchange measurement using a Li-6800; **f** typical gas exchange measurement in a flood rice field. See details in the text

Leaf gas exchange measurement is a direct way to evaluate the photosynthetic capacity of a plant [23]. All leaf gas exchange systems work by enclosing an entire or part of a leaf within a cuvette. The differences in CO_2_ and vapor concentration (generally determined using an infra-red gas analyzer, IRGA) between the ‘reference’ air flow that enters the cuvette and the ‘sample’ air flow that exits the cuvette are used to calculate the rates of photosynthesis (or respiration) and transpiration. By recording other environmental parameters, some photosynthetic parameters, for instance, stomatal conductance and CO_2_ concentration in the intercellular air space, can be calculated [10, 37]. For most of the commercial gas exchange systems, the quantity and quality of the light, flow rate of air, air humidity, temperature, and concentration of atmospheric gases can be controlled to determine the photosynthetic response of the area of leaf contained within the cuvette. Using these systems, light- and CO_2_ response curves can also be estimated to provide photosynthetic mechanistic information [23]. However, photosynthesis is sensitive to environment changes. To obtain the comparable measurements for different genotypes and/or treatments, gas exchange measurements are typically conducted under controlled conditions, requiring a long time for a leaf inside the cuvette to acclimate to the new environmental conditions [15]. In addition, precise controlling of the environment inside cuvettes, especially, temperature and humidity, requires a relatively stable ambient environment; hence, in most case, gas exchange measurement can only be performed within a short period under field conditions. Increasing throughput requires a large number of units, which is very expensive. Furthermore, some crops grow in special conditions, making it very difficult to perform gas exchange measurement in situ. For instance, rice, a staple food of more than half the world’s population, grows in flooded conditions (Fig. 1).

Here we present a fast photosynthesis measurement (FPM) method that reduces the time necessary to measure an instantaneous point gas exchange measurement of rice leaves to less than 2 min, making it possible to screen several hundreds of leaves per day with a single gas exchange system. We finally applied FPM to a F2 population to investigate the genetic variation of photosynthetic traits.

## Results

### Excised vs in situ tillers

**Fig. 2:**
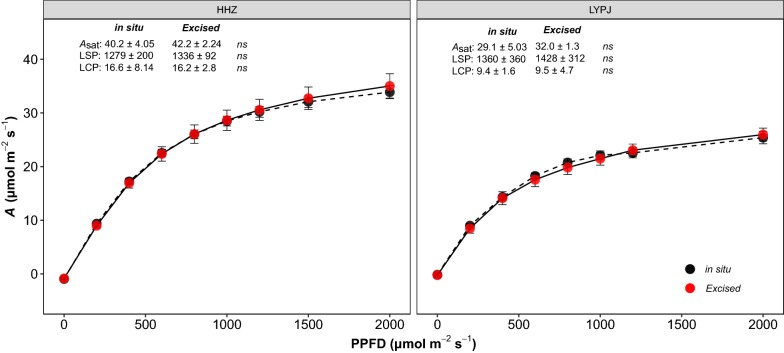
Photosynthetic light-response curves of HHZ and LYPJ measured for excised tillers in the lab conditions and for in situ outdoor plants. *A*_sat_, fitted maximum net photosynthetic rate; LSP, photosynthetic photon flux density at 75% saturation of photosynthesis; LCP, light compensation point. For details about the light response curve fitting, refer to the text. Values show in mean ± SD; *N* = 6; *ns*, no difference between the two methods, *p* > 0.05

**Fig. 3:**
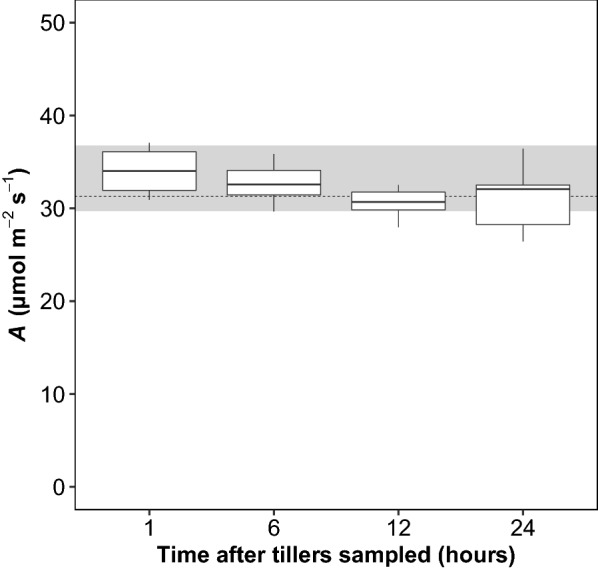
Influence of the storage time of excised tillers on photosynthesis (*A*). All the tillers were sampled in the early morning, and tillers for fast gas exchange measurements were prepared as described in the text. The shaded area and dotted line indicate the interquartile range (IQR) and median value of in situ *A*, respectively. In-situ *A* measured under field conditions in the early morning and using the traditional approach (see details in the text). *N* = 10

Light response curves of excised and in situ tillers were estimated using HHZ and LYPJ grew in the field and in pot conditions, respectively (Fig. 2). The light response curves of excised tillers and in situ tillers were similar in shape, and no difference in photosynthetic rates (*A*) were observed at any light intensity. Moreover, no differences were observed in fitted parameters of light response curves, although the light-saturated photosynthetic rates (*A*_sat_) of excised tillers were slightly elevated in both genotypes. Our results showed that the *A*_sat_ and light compensation point (LCP) of HHZ was higher than LYPJ, but no difference in light intensity was detected under 75% saturated photosynthesis. To investigate the impacts of storage time of excised tillers in the lab on gas exchange measurements, photosynthesis of excised tillers that were maintained in the lab for 1 to 24 h were measured. Although the photosynthetic rate of excised tillers declined with storage time, no significant differences in photosynthetic rate were found between excised tillers from any storage time and in situ plants (Fig. 3).

### Effects of sampling time and illumination time on photosynthesis

**Fig. 4:**
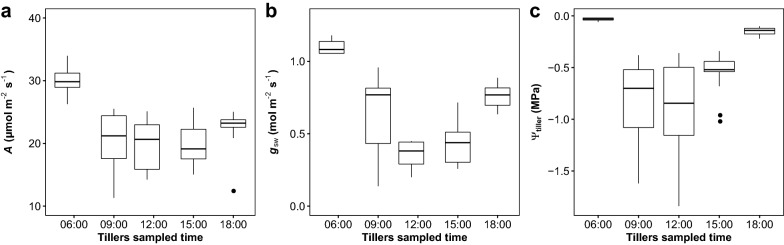
Gas exchange and water potential of HHZ tillers sampled at different times during a day. **a** Photosynthetic rate, **b** stomatal conductance to vapor, and **c** leaf water potential. Tillers for gas exchange measurements were prepared as described in the text and kept in the dark for 1 to 2 h before exposure to light for acclimation. Tillers for water potential measurement were equilibrated in double bags in the dark for 25 min after sampling. *N* = 10

To investigate the influences of sampling time on photosynthesis of excised tillers, the gas exchange of tillers (N = 10) excised at different times during the day was measured. We found that the tillers sampled in the early morning had the highest *A* and stomatal conductance to vapor (*g*_sw_), and moreover, the variabilities of *A* and *g*_sw_ were lower in tillers sampled in early morning and at end of the day (6:00 and 18:00) than those sampled at midday (Fig. 4). Interestingly, the leaf water potential showed the same pattern as *A* and *g*_sw_.

**Fig. 5:**
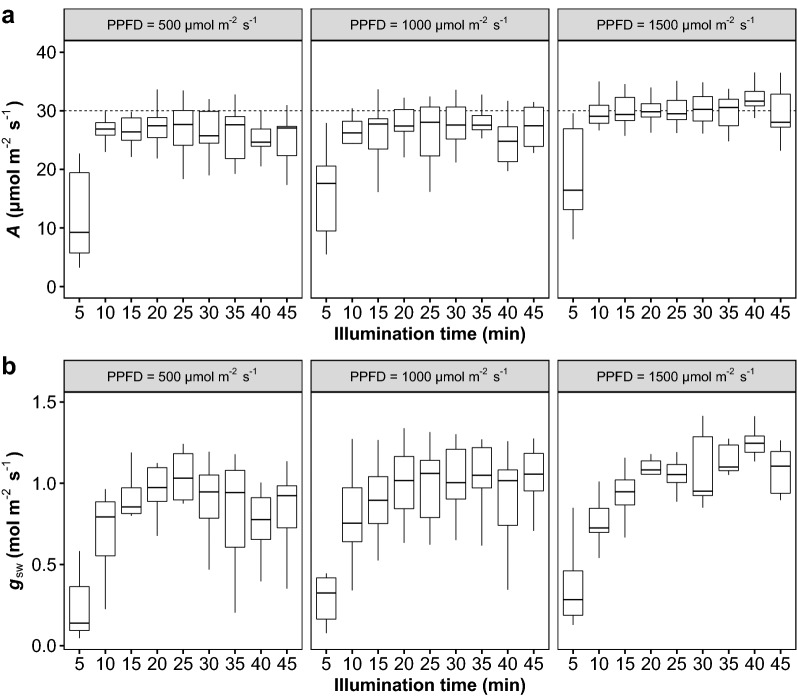
Effects of the illumination time and light intensity on **a** the photosynthetic rate (*A*) and **b** stomatal conductance to vapor (*g*_sw_) of excised tillers. The tillers were sampled in the early morning and kept in the dark before exposure to light. The photosynthetic photon flux density inside the gas exchange cuvette was 1500 µmol m^−2^ s^−1^ for all the measurements. *N* = 10

To test the accommodation time effects, a fast gas exchange measurement was performed to excised tillers with different adaptation times and light intensities combinations in the lab condition. We showed that rice leaves achieved maximum photosynthetic rates within 10 min under all three light intensities (Fig. 5). Unlike photosynthetic rate, more than 20 min were needed for tillers to fully open their stomata. The tillers exposed to 1500 μ mol m^−2^ s^−1^ photosynthetic photon flux density (PPFD) had the highest stabilized photosynthetic rate and stomatal conductance.

### Genetic variation of photosynthesis

**Fig. 6:**
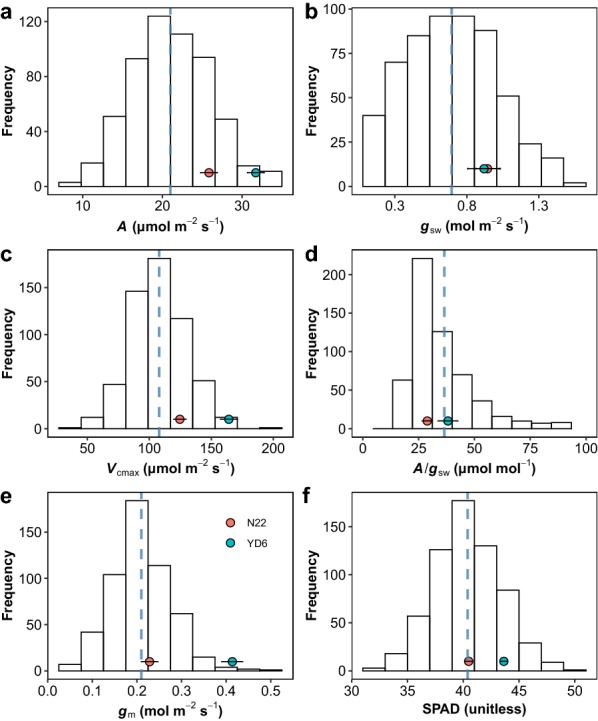
Frequency distributions of the leaf gas exchange parameters and leaf chlorophyll content (**a** photosynthetic rate; **b** stomatal conductance to vapor; **c** maximum rate of carboxylation; **d** leaf intrinsic water use efficiency; **e** mesophyll conductance to CO_2_; **f** leaf chlorophyll content) of 568 F2 lines. Mean values with SDs of the parental lines (*N* = 20) YD6 and N22 are indicated. The dotted line indicates the mean value of F2 lines

**Fig. 7:**
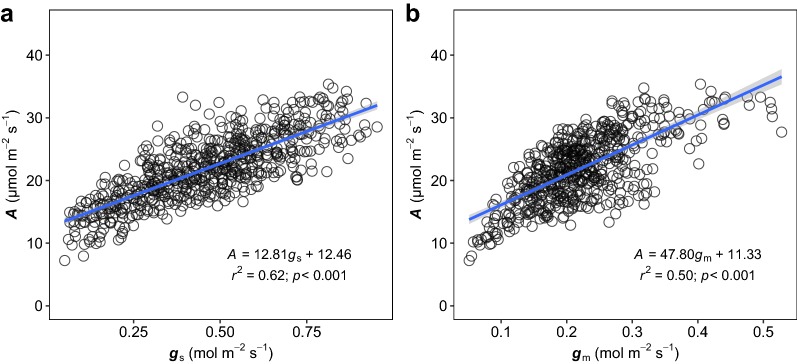
Correlation between the photosynthetic rate (*A*) and **a** stomatal conductance to CO_2_ (*g*_s_) and **b** mesophyll conductance to CO_2_ (*g*_m_) across F2 lines

The genetic variations of gas exchange and chlorophyll content of 568 F2 lines and their parental lines YD6 and N22 were investigated over 2 days using the FPM technique. Among the F2 populations, the *A* and *g*_sw_ varied from 7.2 to 34.76 µmol m^−2^ s^−1^ and 0.081 to 1.53 mol m^−2^ s^−1^, with means of 21.03 µmol m^−2^ s^−1^ and 0.69 mol m^−2^ s^−1^, respectively (Fig. 6). The two parental lines, YD6 and N22, had *A* of 31.7 ± 1.04 and 25.8 ± 1.04 µmol m^−2^ s^−1^, *g*_sw_ of 0.92 ± 0.11 and 0.94 ± 0.08 mol m^−2^ s^−1^, respectively (mean ± standard error (SE), n = 20). Mesophyll conductance (*g*_m_), varied tenfold, from 0.051 to 0.51 mol m^−2^ s^−1^, with a mean of 0.21 mol m^−2^ s^−1^. The two parental lines, YD6 and N22, had *g*_m_ of 0.414 ± 0.024 and 0.228 ± 0.019 mol m^−2^ s^−1^ (mean ± SE), respectively. In this study, the maximum carboxylation efficiency (*V*_cmax_), varied fivefold, from 44.6 to 191.7 µmol m^−2^ s^−1^, with a mean of 107.8 µmol m^−2^ s^−1^. The two parental lines YD6 and N22 had *V*_cmax_ of 164.2 ± 6.9 and 124.7 ± 5.1 µmol m^−2^ s^−1^ (Mean ± SE), respectively. Surprisingly, *g*_sw_, *g*_m_ and *A*/*g*_sw_ were skewed from the normal distribution (Additional file 1: Table S1, Additional file 2: Figure S1; Fig. 6). Gas exchange parameters (*A*, *g*_sw_, *V*_camx_, *g*_m_, *A*/*g*_sw_) were significantly correlated with each other, except relationship between *V*_cmax_ and *A*/*g*_sw_ (Additional file 3: Table S2). *A* was strongly related to *g* (*A* = 12.81 *g* + 12.46; *r*^2^ = 0.62; *p *< 0.001) and *g*_m_ (*A* = 47.80 *g*_m_ + 11.33; *r*^2^ = 0.50; *p *< 0.001) across rice genotypes (Fig. 7). Both of *A* and *V*_cmax_ were significantly related to SPAD.

## Discussion

Instantaneous point measurement of leaf gas exchange is the most widely used technique when assessing the photosynthetic capacity of plants under experimental or natural conditions [13, 26, 43]. Point measurements can be performed under ambient or set conditions of CO_2_ concentration, light intensity, temperature and humidity within the leaf cuvette of the gas exchange system. As leaves do not need to acclimate to new environmental conditions inside the cuvette, the use of ambient cuvette conditions in the field may be beneficial in terms of an increased speed of measurement [39]. However, variation in environmental conditions, such as light intensity and air temperature, influence consistency between measurements [3, 18, 25]. Instantaneous point measurements under set controlled cuvette conditions generally require a long time for leaves to acclimate to the new conditions within the leaf cuvette [46]. A maximum rate of 30 measurements per day per gas exchange unit has been practically confirmed in environmentally controlled growth chambers [1], and much fewer measurements can be accomplished in field conditions due to the heterogeneity of environmental factors over the day and/or circadian regulation [29]. In fact, it is preferable to conduct gas exchange measurements in a short-term period (i.e., between 9:00 and 11:30 am) on clear sunny days under field conditions, which restricts the throughput and repeatability of gas exchange measurements in traditional approaches. Here, more than 300 robust gas exchange measurements were performed per day per LI-6800. Although FPM is a destructive approach, it can overcome the diurnal variations and/or environmental changes that introduce errors in light-saturated photosynthetic capacity investigations (Fig. 3), and it is possible to perform the measurements for 12 h or more hours per day. The FPM technique’s success is mainly attributed to that it allows the leaves to acclimate to saturating light after darkness equilibrium under environmentally controlled lab condition.

Light response and CO_2_ response curves are two widely used measurements that provide mechanistic information, such as quantum yield, daytime respiration and photosynthetic biochemical limitations [32, 44, 45]. However, these measurements are time-consuming: 1 to 3 curves (depending on the system, number of steps and ambient conditions) per hour under field conditions. Similar to the instantaneous point measurements under set controlled cuvette conditions, delays for acclimation to the controlled environment inside the cuvette represent the bottleneck of the throughput. We achieved an average throughput of 5.4 light response curves per hour—3 times greater than the 1.8 light response curves per hour possible using the traditional method in the rice field, but with the same data quality (Fig. 2). Importantly, the gas exchange measurements can only be conducted between 9:00 and 12:00 on sunny days using the traditional approach, due to the midday depression of photosynthesis [27] and/or the heterogeneity of environmental conditions throughout the day. In contrast, whole day measurements are possible using the FPM approach (Fig. 3).

### Answers and further research questions arising from the FPM technique

In this study, we also observed some interesting biological behaviors which were generally ignored in the past. First, we found that the gas exchange of excised tillers was highly dependent on the sampling time (Fig. 4). The photosynthesis and stomatal conductance were significantly lower for the tillers sampled during the day than in early morning. The decline of the gas exchange rate could be, at least partly, explained by the changes in plant water potential. When the tiller water potential is quite low (− 1.9 to − 0.45 MPa in this study), the released tension of xylem caused by tiller excision may introduce cavitation in the xylem, therefore restricting water transportation in the plant [36]. Although the excised tillers were cut under water, cavitation might be caused by the thin gas film on the razor blade surface. Refilling under low transpiration conditions is considered an efficient approach to remove cavitation in the xylem; however, recent studies have suggested that embolic xylem is unable to recover even when kept in darkness [7, 17]. If cavitation is the casual factor, then current approaches for leaf and stem hydraulic capacity estimation are dubious, at least for rice, as tillers are typically sampled during the day. Unfortunately, in this study, cavitation was not estimated because it was not tightly related to the key questions that arose in this study, necessitating further estimations.

Second, gas exchange measurements were affected by the light intensity gradients between the inside and outside of the gas exchange cuvette (Fig. 5). The leaves that had adapted to 1000 or 500 µmol m^−2^ s^−1^ photosynthetic photon flux density had a low *A* compared with leaves that had adapted to 1500 µmol m^−2^ s^−1^ photosynthetic photon flux density. One possible explanation for this finding is that plants may have the ability to optimize their photosynthetic enzymes to acclimate to a given ambient light condition, and a long time is required to acclimate to a new light environments [20, 30]. Another candidate mechanism may be related to the light dependence of mesophyll conductance, which has been widely observed in the past decades [4, 5, 35, 42]. Again, although those candidate mechanisms were not investigated in the current study, this information is important to improve understanding of the light responses of photosynthesis and to measure gas exchange accurately, as current light-saturated photosynthesis measurements are typically estimated under dynamic light conditions.

### Limitations of FPM and potential improvements

Just as traditional gas exchange approaches are not optimized for throughput, FPM is not optimized for many experimental situations. First, the FPM approach is not suitable to estimate gas exchange for water-controlled plants. Second, stomatal conductance estimated using the FPM approach was slightly higher compared with the values estimated using the traditional in situ approach. Stomatal conductance has been suggested to be limited by the leaf water potential; however, the excised tillers assimilated water through the cut surface of the stem where the water potential was nearly zero and therefore potentially increased the leaf water potential. However, the water potential issue could be partly solved using plural osmotica (i.e. artificial xylem sap) to simulate the natural stem water potential. Third, FPM is likely unable to estimate the natural diurnal variations of gas exchange in the current stage because leaves are acclimated to the environmentally controlled lab conditions. Finally, due to lack of root, the functions of the root systems in gas exchange cannot be evaluated.

### Genetic variation of rice photosynthesis

In this study, by using the FPM technique, we investigated the rice genetic variations of photosynthetic traits, including *A*, *g*_s_, *g*_m_ and *V*_cmax_, across 568 F2 lines. Although quantitative trait locus (QTL) analysis was not performed, QTL and/or genome-wide association study (GWAS) analysis to photosynthetic traits are possible using the FPM technique to phenotype photosynthetic traits. We did not perform QTL analysis in the present study due to the absence of: (1) a genetic map for the population, currently and (2) biological replicates for each line because the F2 individuals are heterozygous.

Six gas exchange related traits were detected and most of their distribution were approximately (*p* > 0.01; Additional file 1: Table S1) normal distribution except *A*/*g*_sw_. The negatively skewed ‘*A*/*g*_sw_’ is mainly because of the positively skewed *g*_sw_ distribution. Great variations among F2 lines were observed, but only a few genotypes had a slightly higher the photosynthetic rate than the parent YD6. As YD6 has been the most widely used parental line for super rice breeding in China over the past decades, its photosynthetic assimilation rate may have already been selected by breeders. Moreover, both stomatal and mesophyll conductance were tightly correlated to the photosynthetic rate among genotypes (Fig. 7). Correlations between conductance and photosynthetic rate are often observed (see a recent meta-analysis [40] and references therein), and the CO_2_ concentration within the chloroplast is considered a target trait for a high rate of photosynthesis [11].

## Conclusions

The methodology described herein elucidates a fast, high throughput approach, FPM, for phenotyping photosynthetic capacity in a major crop species under lab conditions. We demonstrated that excised tillers can be used as a robust proxy for leaf gas exchange estimation. Furthermore, we discussed the advantages and disadvantages of FPM. Finally, we investigated the genetic variations of photosynthetic traits across 568 F2 lines using the FPM technique and discussed the logistics of screening several hundred samples per day per instrumental unit using FPM to contribute a wealth of photosynthetic phenotypic data and improve selection power in large populations of rice, with the ultimate aim of improving yield through improved photosynthesis.

## Methods

### Plant materials

*Oryza sativa*, cv Huanghuazhan (HHZ) and Liangyoupeijiu (LYPJ) were used to establish the FPM. HHZ is a widely cultivated local inbred variety and LYPJ is a famous hybrid rice cultivar in China. HHZ seedlings, 25 days after sowing in the nursery, were transplanted into a paddy field (Huazhong Agricultural University, Wuhan, Hubei, China) at a plant density of 33.3 × 33.3 cm. The compound fertilizer (N: P_2_O_5_: K_2_O = 16%: 16%: 16%; Batian Ecological Engineering Limited, Shenzhen, China) at a rate of 375 kg ha^−1^ was applied to the field 2 days before transplanting, and urea at a rate of 100 kg ha^−1^ was applied 2 weeks after transplanting. Herbicides, pesticides and germicides were applied regularly to avoid any stress. LYPJ plants were grown in outdoor pot conditions on Huazhong Agricultural University campus. Twenty-two-days-old seedlings of LYPJ were transplanted into 11 L pots containing 10 kg dry soil mixed with 0.51 g urea, 1.96 g superphosphate and 0.28 g potash muriate. Nitrogen topdressing was applied 2 weeks after transplanting (0.39 g urea per pot). Each pot contained three seedlings, and there were 48 pots. The visible water layer was maintained during the experiment by daily watering.

The F2 populations, derived from a reciprocal cross between Yangdao 6 (YD6) and N22, were grown under field conditions to access the throughput of PFM. YD6 is a parent of many super rice cultivars that have been released in past decades in China, and N22 is an abiotic stress tolerance genotype [16, 31]. Our preliminary experiment results showed that *A*_sat_ of YD6 was higher than N22. The F2 populations were grown in the same field plot as HHZ and with equivalent all management practices.

### Tiller preparation for FPM in the lab

The tiller preparation process is shown in Fig. 1. In short, the main tillers at the panicle initiation stage were first cut using a new razor blade under paddy water, and the cut surface of the tillers was immediately transferred to airless distilled water and cut a second time under water. The tillers were then covered with double black bags and transferred to the lab (approximately 1000 m from the experimental field). In the lab, all the non-target leaves were removed from the stem to ensure perfect illumination of the target leaves, and the tillers were recut under airless distilled water. Finally, the cut surface of a single tiller was transferred to a 25-ml glass tube filled with airless distilled water under water and maintained in the lab (air temperature, 28 °C; relative humidity, 50%; PPFD < 10 µmol m^−2^ s^−1^). All the tillers, except for the sample time test samples, were harvested from the plants prior to 06:40 (dawn time between 05:40 and 05:51 during the experiment). For the genetic variation experiment, 295 F2 tillers of YN (YD6 × N22), 273 F2 tillers of NY (N22 × YD6) and ten tillers of each parent were harvested before 06:40 from the field. Before performing the gas exchange measurements, the target leaf area was illuminated with 1500 µmol m^−2^ s^−1^ (estimated using Li190SB, Li-COR Inc., Lincoln, NE, USA) for 30 min to achieve a photosynthetic stable status using a LED light source (Weichuang Electronic Technology Limit, Wuhan, China).

### Gas exchange measurements

Gas exchange was estimated using a LI-6800 (Li-COR Inc., Lincoln, NE, USA). To minimize gradients between lab ambient conditions and inside the cuvette of the gas exchange system, the sample CO_2_ concentration, relative humidity and leaf temperature inside the cuvette were set to 400 µmol mol^−1^, 65% and 28 °C, respectively. Light response (AQ) curves of HHZ and LYPJ were generated on excised tillers in the lab conditions, and in situ plants in pots or field conditions. Based on the light response curve results, the PPFD inside the LI-6800 cuvette was set to 1500 µmol m^−2^ s^−1^ for photosynthesis measurements. As a great number of plants are required to test sampling time and illumination time effects, only the HHZ plants grown in the field condition were used in these tests. Leaves were held in the cuvette until the photosynthesis values were stable, i.e., ‘steady state’, which generally occurred rapidly (~ 30 s) due to the similarity of the conditions inside and outside the leaf chamber. Typically, measurements were collected if the changes in photosynthetic rate were less than 0.5% over 1 min (Fig. 1). Outdoor in situ gas exchange estimation was conducted between 8:30 and 11:30 using the (standard) traditional approach. The environmental conditions inside the cuvettes were set as above, and before recording the gas exchange data, at least 20 min were generally required to achieve a stable gas exchange measurement (photosynthetic rate variation of less than 0.5% in 1 min).

For population materials, leaf gas exchange and chlorophyll fluorescence were measured simultaneously. The electron transport rate (*J*) was then calculated as follows:

$$J = \varPhi_{\text{PSII}} \cdot {\text{PPFD}} \cdot \alpha \beta ,$$

where, Φ_PSII_ is the actual photochemical efficiency of photosystem II, *α* is the leaf absorptivity and *β* is the partitioning of absorbed quanta between photosystems II and I. In the current study, the *αβ* value of 0.44 was used based on the previous studies [38, 41].

The variable *J* method [14] was used to calculate *g*_m_:

$$g_{\text{m}} = \frac{A}{{C_{i} - \frac{{\Gamma^{*} \left( {J + 8\left( {A + R_{\text{d}} } \right)} \right)}}{{J - 4\left( {A + R_{\text{d}} } \right)}}}},$$

where *A* is the photosynthetic rate at PPFD of 1500 μmol m^−2^ s^−1^, *C*_i_ is the CO_2_ mol fraction in the intercellular air space, Γ^*^ represents the CO_2_ compensation point in the absence of mitochondrial respiration and *R*_d_ is the daytime respiration rate. Typical values of 40 μmol mol^−1^ and 1 μmol m^−2^ s^−1^ were used for Γ^*^ and *R*_d_, respectively [38]. For each data point generated, we checked whether it met the criterion (10 > *dC*_c_/*dA *> 50) [14].

The ‘one-point’ method [9] was used to calculate *V*_cmax_:

$$V_{\text{cmax}} = A\left( {\frac{{C_{\text{i}} + K_{\text{m}} }}{{C_{\text{i}} - \Gamma^{*} }} - 0.015} \right),$$

where *K*_m_ is apparent Michaelis constant for carboxylation which was calculated:

$$K_{\text{m}} = K_{\text{c}} \left( {1 + \frac{{O_{\text{i}} }}{{K_{\text{o}} }}} \right)$$

where *K*_c_ is Michaelis constant for carboxylation, and *K*_O_ is Michaelis constant for oxygenation, *O*_i_ is oxygen content in the intercellular air space. We chiefly use values taken from [2] calibrated to 25 °C.

### Chlorophyll content and leaf water potential

In this study, the dynamic of water potential during the day was estimated using a pressure chamber (Soil Moisture Equipment Crop., Santa Barbara, Ca., USA). Tillers were double bagged shortly after sampling and kept in a cool box for 25 min for equilibration. Chlorophyll content was measured using SPAD 502 (Konica Minolta, Japan). Eight points were measured in 5-cm longitudinal distance in the middle of the leaf after the gas exchange measurement, and the values were averaged.

### Data analysis

Light response curve parameters, including the maximum net photosynthetic rate (*A*_sat_), light compensation point (LCP) and PPFD at the 75% saturation photosynthetic rate (LSP) were fitted using the nonrectangular hyperbola–based model [21]:

$$A = \frac{{\varPhi \times {\text{PPFD}} + A_{\text{gmax}} - \sqrt {(\varPhi \times {\text{PPFD}} + A_{\text{gmax}} )^{2} - 4\theta \times \varPhi \times {\text{PPFD}} \times A_{\text{gmax}} } }}{4\theta } - R_{\text{n}}$$

where Φ is the quantum yield at PPFD = 0 µmol (photon) m^−2^ s^−1^, *A*_gmax_ is the maximum gross photosynthetic rate, *θ* is the convexity factor, and *R*_n_ is dark respiration. The model was fitted to the data using the Orthogonal Nonlinear Least-Squares Regression (*onls*) function. Other analyses and plots were conducted using the *tidyverse* R package. All analyses were performed in R 3.6.0.

## Supplementary information

**Additional file 1: Table S1.** Test of normality (Shapiro–Wilk) for photosynthetic traits of F2 populations. The full name and units of the traits are shown in abbreviations list.

**Additional file 2: Figure S1.** Normal Q–Q plot of photosynthetic traits.

**Additional file 3: Table S2.** Correlations between the photosynthetic traits of F2 populations. The full name and units of the traits are shown in abbreviations list. The correlations were estimated by the linear model. *Significant at 5% level.

## Data Availability

The datasets used and/or analyzed during the current study are available from the corresponding author on reasonable request.

## Abbreviations

FPM: fast photosynthesis measurement
HHZ: *Oryza sativa* cultivar Huanghuazhan
LYPJ: *Oryza sativa* cultivar Liangyoupeijiu
YD6: *Oryza sativa* cultivar Yangdao 6
PPFD: photosynthetic photo flux density
QTL: quantitative trait loci analysis
Φ_PSII_: photochemical efficiency of photosystem II
α: light absorptivity of leaf
*β*: distribution of absorbed quanta between photosystems II and I
*g*_m_: mesophyll conductance to CO_2_ diffusion (mol m^−2^ s^−1^)
*A*: net photosynthetic rate (µmol m^−2^ s^−1^)
Γ^*^: CO_2_ compensation point in the absence of mitochondrial respiration (µmol mol^−1^)
*J*: electric transport rate (µmol m^−2^ s^−1^)
*C*_i_: CO_2_ mol fraction in the intercellular air space (µmol mol^−1^)
*R*_d_: daytime respiration rate (µmol m^−2^ s^−1^)
*V*_cmax_: maximum carboxylation efficiency (µmol m^−2^ s^−1^)
*K*_m_: apparent Michaelis constant for carboxylation
*K*_c_: Michaelis constant for carboxylation
*O*_i_: oxygen content in the intercellular air space (µmol mol^−1^)
*K*_o_: Michaelis constant for oxygenation
*g*_sw_: stomatal conductance to vapor diffusion (mol m^−2^ s^−1^)
*g*_s_: stomatal conductance to CO_2_ diffusion (mol m^−2^ s^−1^)
LCP: light composition point (µmol m^−2^ s^−1^)
LSP: PPFD at 75% saturation photosynthetic point (µmol m^−2^ s^−1^)
Φ: quantum yield at PPFD = 0 µmol (photon) m^−2^ s^−1^
*A*_gmax_: maximum gross photosynthetic rate (µmol m^−2^ s^−1^)
*θ*: convexity factor
*R*_n_: dark respiration rate (µmol m^−2^ s^−1^)
*A*_sat_: maximum net photosynthetic rate in light response curve (µmol m^−2^ s^−1^)
