# Supplementary material for: Fast photosynthesis measurements for phenotyping photosynthetic capacity of rice
Source: Plant Methods. 2020 Jan 24;16:6. doi: 10.1186/s13007-020-0553-2 (PMC6979334; doi:10.1186/s13007-020-0553-2)
Supplement: Supplementary file 1 — Additional file 1: Table S1. Test of normality (Shapiro–Wilk) for photosynthetic traits of F2 populations. The full name and units of the traits are shown in abbreviations list. [file 13007_2020_553_MOESM1_ESM.docx]

**Supplemental materials**

**Fast photosynthesis measurements for phenotyping photosynthetic capacity of rice**

**Table S1.** Test of normality (Shapiro-Wilk) for photosynthetic traits of F2 populations. The full name and units of the traits are shown in abbreviations list.

| **Traits** | **Shapiro-Wilk statistic** | ***p*** |
| --- | --- | --- |
| *A* | **0.997** | **0.636** |
| *g*_sw_ | 0.985 | 0.036 |
| *V*_cmax_ | **0.996** | **0.155** |
| *A*/*g*_sw_ | 0.774 | <0.001 |
| *g*_m_ | 0.986 | 0.030 |
| SPAD | **0.997** | **0.352** |
