# Supplementary material for: Fast photosynthesis measurements for phenotyping photosynthetic capacity of rice
Source: Plant Methods. 2020 Jan 24;16:6. doi: 10.1186/s13007-020-0553-2 (PMC6979334; doi:10.1186/s13007-020-0553-2)
Supplement: Supplementary file 2 — Additional file 2: Figure S1. Normal Q–Q plot of photosynthetic traits. [file 13007_2020_553_MOESM2_ESM.docx]

**Supplemental materials**

**Fast photosynthesis measurements for phenotyping photosynthetic capacity of rice**


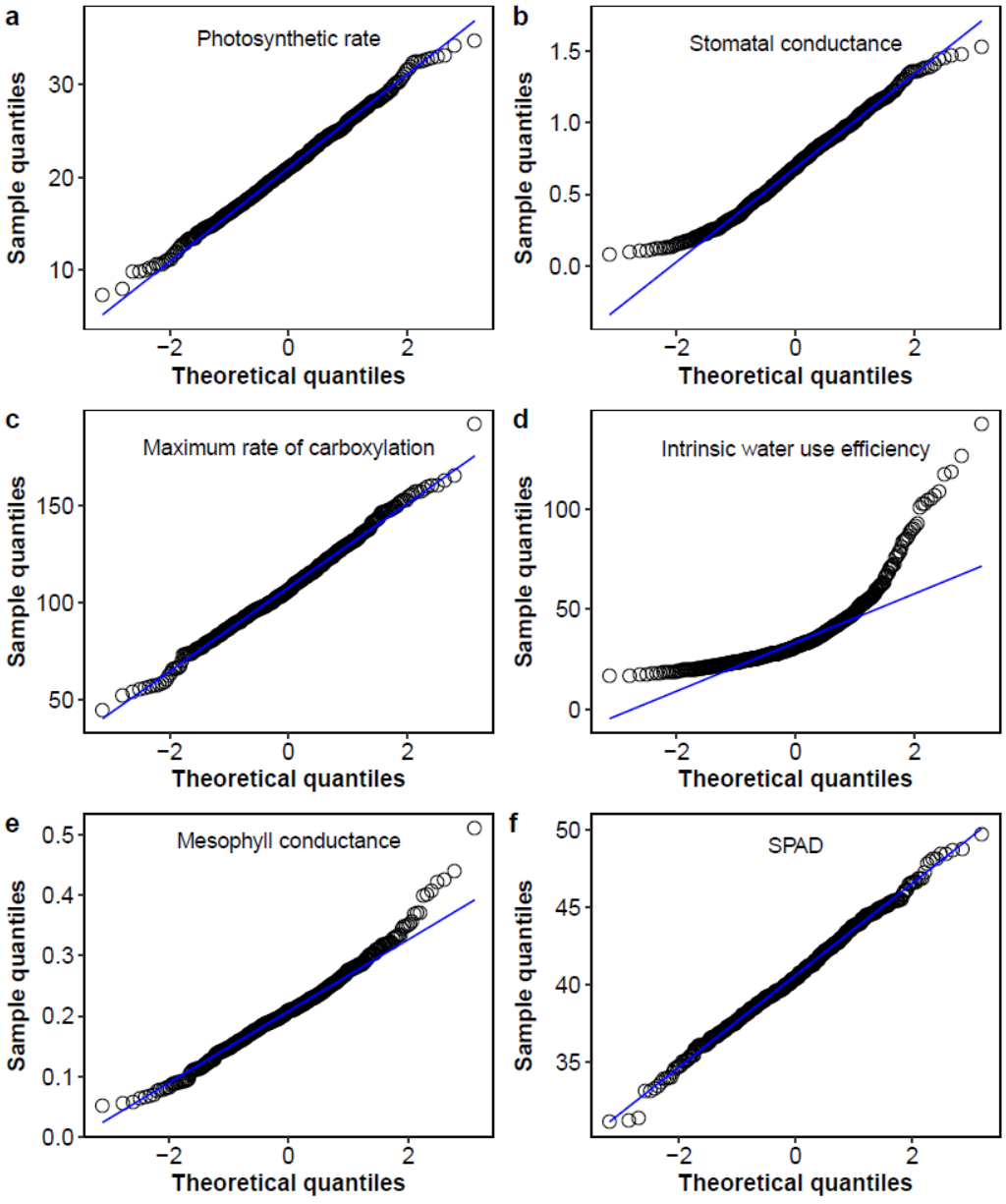


**Figure S1** Normal Q-Q plot of photosynthetic traits.
