# Supplementary material for: Fast photosynthesis measurements for phenotyping photosynthetic capacity of rice
Source: Plant Methods. 2020 Jan 24;16:6. doi: 10.1186/s13007-020-0553-2 (PMC6979334; doi:10.1186/s13007-020-0553-2)
Supplement: Supplementary file 3 — Additional file 3: Table S2. Correlations between the photosynthetic traits of F2 populations. The full name and units of the traits are shown in abbreviations list. The correlations were estimated by the linear model. *Significant at 5% level. [file 13007_2020_553_MOESM3_ESM.docx]

**Supplemental materials**

**Fast photosynthesis measurements for phenotyping photosynthetic capacity of rice**

| **Table S2.** Correlations between the photosynthetic traits of F2 populations. The full name and units of the traits are shown in abbreviations list. The correlations were estimated by the linear model. * Significant at 5% level. | | | | | |
| --- | --- | --- | --- | --- | --- |
|  | ***A*** | ***g*_sw_** | ***V*_camx_** | ***g*_m_** | ***A*/*g*_sw_** |
| ***g*_sw_** | 0.79* |  |  |  |  |
| ***V*_cmax_** | 0.86* | 0.44* |  |  |  |
| ***g*_m_** | 0.71* | 0.47* | 0.70* |  |  |
| ***A*/*g*_sw_** | -0.47* | -0.77* | 0.04 | -0.19* |  |
| **SPAD** | 0.23* | 0.14* | 0.28* | 0.27* | 0.09 |
|  | | | | | |
